# Welcome to pneumonia Volume 2

**DOI:** 10.15172/pneu.2013.2/242

**Published:** 2013-01-10

**Authors:** Allan W. Cripps

**Affiliations:** Griffith Health, Griffith University, Gold Coast Campus, Queensland, 4222 Australia

Starting a new journal is always challenging as new IT systems are put in place. Thank you to all our readers, our reviewers, those who submitted manuscripts and those whose manuscripts were published, for their patience and generosity in thought.

In the first six months of the journal’s existence, we published three quality papers. Over this time, the journal has been visited from every continent and 21 countries, with nearly 70% of these being from new visitors. The average visit duration is a little over 4 minutes. These are very exciting statistics for a journal in its infancy as **pneumonia** is.

In Volume 1, papers were published from Malawi, examining oxygen supply and demand in a large teaching hospital setting; Australia concluding that qPCR analysis of sera has potential to aid the aetiological diagnosis of pneumonia; and France which concluded that viral co-infection was observed mainly in patients with severe pneumonia and that RT-PCR substantially improved pathogen detection.

**pneumonia** is the only journal that is exclusively focused on pneumonia. Welcome to Volume 2.

